# A gelatin hydrogel nonwoven fabric improves outcomes of subcutaneous islet transplantation

**DOI:** 10.1038/s41598-023-39212-4

**Published:** 2023-07-24

**Authors:** Norifumi Kanai, Akiko Inagaki, Yasuhiro Nakamura, Takehiro Imura, Hiroaki Mitsugashira, Ryusuke Saito, Shigehito Miyagi, Kimiko Watanabe, Takashi Kamei, Michiaki Unno, Yasuhiko Tabata, Masafumi Goto

**Affiliations:** 1 Department of Surgery, Tohoku University Graduate School of Medicine, Sendai, 980-0872 Japan; 2 Division of Transplantation and Regenerative Medicine, Tohoku University Graduate School of Medicine, Sendai, 980-8575 Japan; 3 Division of Pathology, Graduate School of Medicine, Tohoku Medical and Pharmaceutical University, Sendai, 983-8536 Japan; 4 Laboratory of Biomaterials, Department of Regeneration Science and Engineering, Institute for Life and Medical Sciences (LiMe), Kyoto University, Kyoto, 606-8507 Japan

**Keywords:** Extracellular matrix, Preclinical research

## Abstract

Subcutaneous islet transplantation is a promising treatment for severe diabetes; however, poor engraftment hinders its prevalence. We previously reported that a recombinant peptide (RCP) enhances subcutaneous islet engraftment. However, it is impractical for clinical use because RCP must be removed when transplanting islets. We herein investigated whether a novel bioabsorbable gelatin hydrogel nonwoven fabric (GHNF) could improve subcutaneous islet engraftment. A silicon spacer with or without GHNF was implanted into the subcutaneous space of diabetic mice. Syngeneic islets were transplanted into the pretreated space or intraportally (Ipo group). Blood glucose, intraperitoneal glucose tolerance, immunohistochemistry, CT angiography and gene expression were evaluated. The cure rate and glucose tolerance of the GHNF group were significantly better than in the control and Ipo groups (*p* < 0.01, *p* < 0.05, respectively). In the GHNF group, a limited increase of vWF-positive vessels was detected in the islet capsule, whereas laminin (*p* < 0.05), collagen III and IV were considerably enhanced. TaqMan arrays revealed a significant upregulation of 19 target genes (including insulin-like growth factor-2) in the pretreated space. GHNF markedly improved the subcutaneous islet transplantation outcomes, likely due to ECM compensation and protection of islet function by various growth factors, rather than enhanced neovascularization.

## Introduction

Pancreatic islet transplantation has been established as a treatment option for severe type 1 diabetes^1^. Although islet transplantation is less invasive, and thus more attractive to patients than pancreas transplantation, there are several issues to be resolved^2^, including the need for multiple donors to cure a single patient and insufficient long-term outcomes^3^. In the current practice of islet transplantation, the intraportal injection of islet grafts is considered to be the gold standard worldwide. However, this approach is associated with several problems, including rapid and severe islet destruction due to strong innate immune reactions in the liver^4–7^, complications such as hemorrhage and portal vein embolism^8,9^, and difficulty in removing transplanted islet grafts when needed in the case of stem cell-derived beta cell replacement. Thus, more ideal transplant sites are strongly desired in the field of islet transplantation.

The subcutaneous space is undoubtedly a candidate site^10^. Indeed, subcutaneous islet transplantation has several advantages, including minimal invasiveness and easy accessibility for the islet grafts, which makes it possible to monitor^11^ and/or remove islet grafts if needed. However, poor vascularization has long been regarded as major drawback in this transplant site^12–14^. Recently, several groups have reported that the compensation of extracellular matrices (ECM) is also crucial for successful subcutaneous transplantation^15,16^.

In fact, we previously reported that a recombinant peptide (RCP: alpha-1 sequence of recombinant collagen type I supplemented with 12 RGD [Arg-Gly-Asp] motifs in 1 molecule) was effective for improving islet engraftment in subcutaneous transplantation, despite the fact that the newly-constructed vessels surrounding the islet grafts were not as enhanced as we expected^17^. Although RCP was effective for subcutaneous transplantation in experimental models, one drawback was to remove RCP when islets are transplanted into the pretreated subcutaneous space, since it is not completely absorbed at least in the murine model. As a result, at least some parts of the newly-compensated ECM and/or vessels induced by RCP might be destroyed. Thus, other types of scaffolds with similar effectiveness to RCP, which also has the feature of being replaced by host cells, is expected to be more preferable for this purpose.

A gelatin hydrogel nonwoven fabric (GHNF), which was fabricated by a blow spinning method of gelatin solution, was reported to work as a functional scaffold for multilayered cell sheets^18^. We speculate that ECM produced by a non-woven fabric structure of GHNF may be similar to that in the human body. Of particular interest, GHNF is bioabsorbable; thus, it does not need to be removed when transplanting islet cells. Furthermore, GHNF was shown to effectively attract cells from adjacent tissues into its body, most likely due to its nonwoven fabric structure^18^, suggesting that it can be completely replaced by host tissues. Given that gelatin itself has been reported to enhance ECM^19^ and/or angiogenesis^20^, GHNF can be expected to be preferable substitute for RCP.

Therefore, in the present study, we investigated whether GHNF could improve subcutaneous islet engraftment in comparison to the current standard method of intraportal islet transplantation.

## Results

### Macroscopic pathology

**Figure 1:**
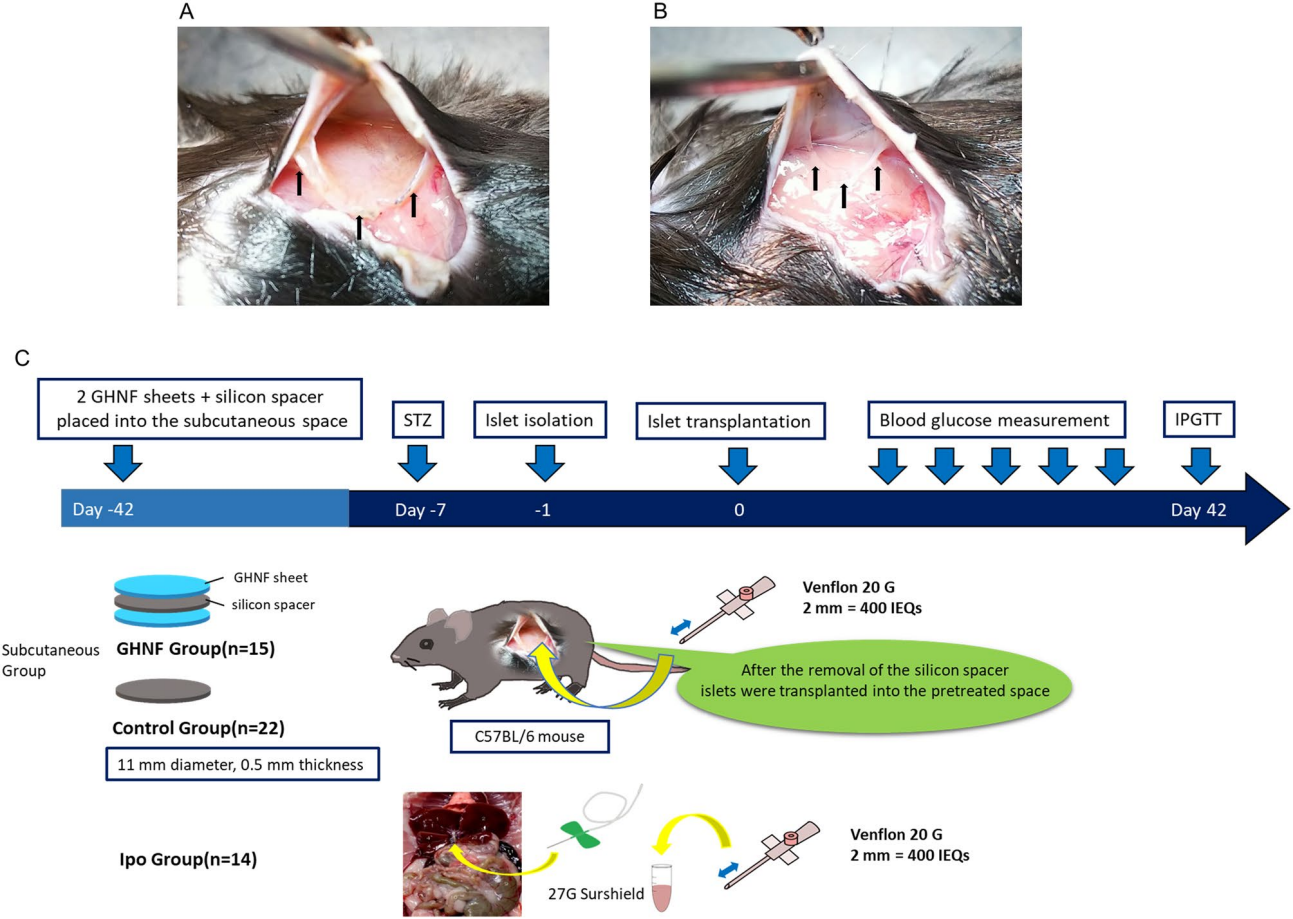
Macroscopic pathology and the scheme of experimental design. Representative photos of the macroscopic pathology of subcutaneous tissues in diabetic mice at 6 weeks after pretreatment in the GHNF (**A**) and control (**B**) groups are shown. The capsule surrounding the silicon spacer in the GHNF group appeared to be thicker than that in the control group (black arrows). No bleeding or inflammation were observed in either group. The scheme of experimental design (**C**).

After pretreatment, the capsule surrounding the silicon spacer at the subcutaneous transplant site in the GHNF group appeared to be thicker than that in the control group. No bleeding or inflammation were observed in either group (Fig. 1A,B).

### The comparison of islet engraftment after marginal islet mass transplantation among the GHNF, control and Ipo groups

**Figure 2:**
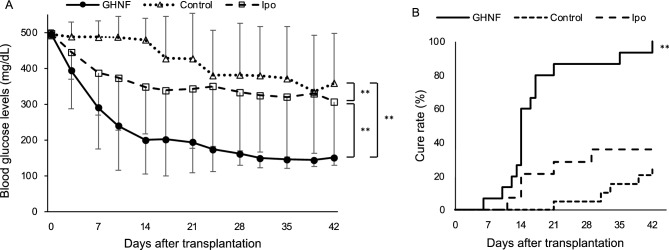
The outcome of islet engraftment after marginal islet mass transplantation (400 IEQs). (**A**) The changes in the blood glucose levels after islet transplantation in the GHNF (filled circle, n = 15), control (open triangle, n = 22), and intraportal transplantation (Ipo) groups (open square, n = 14). The GHNF group demonstrated significantly better glucose changes than the control and Ipo groups (**p < 0.01). (**B**) The cure rate curve of diabetic mice after islet transplantation in each group. The cure rate at 42 days after islet transplantation in the GHNF group (100%) was significantly higher than that in the control (22.7%) and Ipo (35.7%) groups (**p < 0.01).

Blood glucose values after islet transplantation in the GHNF group (n = 15) were significantly lower than those in the control (n = 22) and Ipo (n = 14) groups (*p* < 0.01) (Fig. 2A). The cure rate of diabetic mice at 42 days after islet transplantation in the GHNF group was significantly higher than that in the control and Ipo groups (100% [15/15] vs. 22.7% [5/22] vs. 35.7% [5/14], respectively, *p* < 0.01) (Fig. 2B).

### Intraperitoneal glucose tolerance test

**Figure 3:**
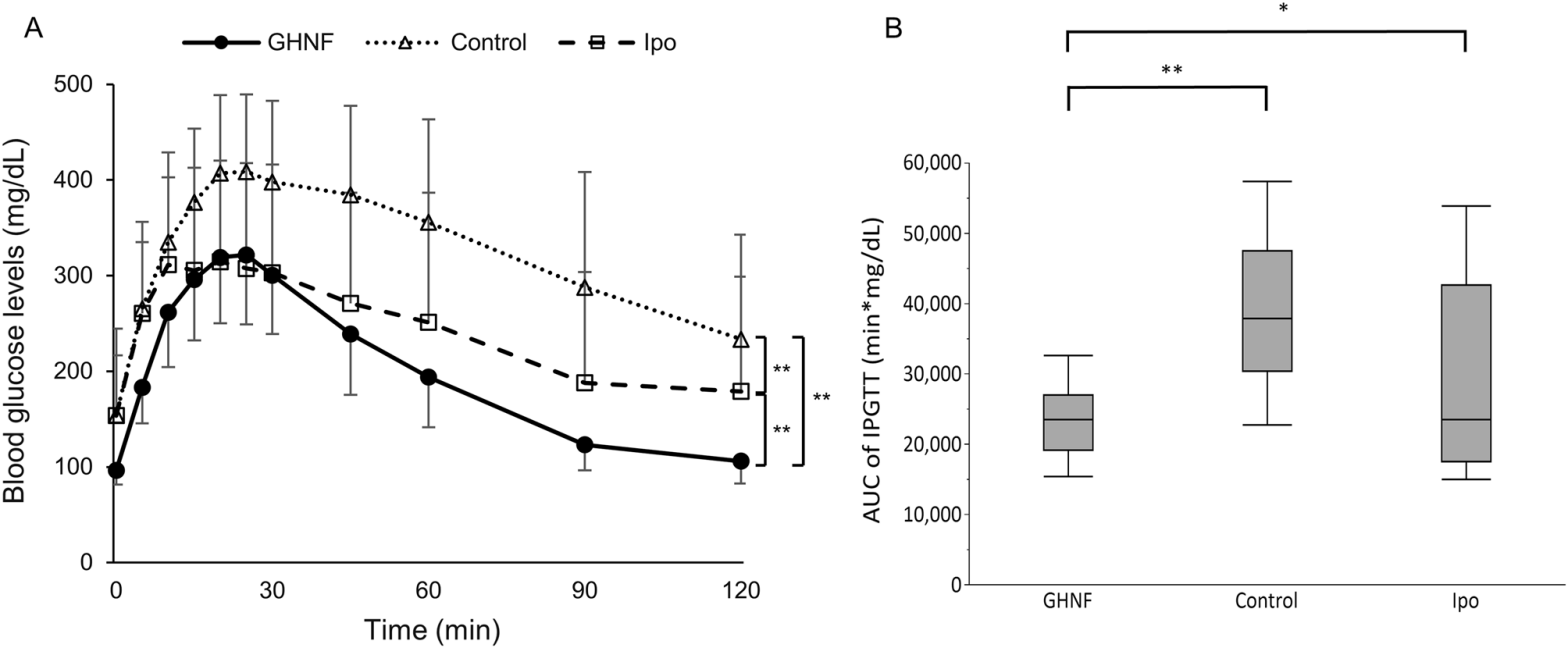
The glucose tolerance profiles of the GHNF, control and Ipo groups. (**A**) The results of the intraperitoneal glucose tolerance test (IPGTT) in the GHNF (filled circle, n = 15), control (open triangle, n = 18), and Ipo groups (open square, n = 12) at approximately 42 days after islet transplantation. (**B**) The area under the curve (AUC) of the IPGTT in each group is shown. The AUC in the GHNF group was significantly lower than that in the control and Ipo groups (*p < 0.05, **p < 0.01).

The intraperitoneal glucose tolerance test (IPGTT) only showed a normal curve pattern in the GHNF group (Fig. 3A). The area under the curve (AUC) in the GHNF group was significantly lower than that in the control and Ipo groups (23,342 ± 4586 vs. 39,247 ± 10,705 vs. 28,961 ± 13,285, respectively, *p* < 0.05) (Fig. 3B).

### Immunohistochemical analyses

**Figure 4:**
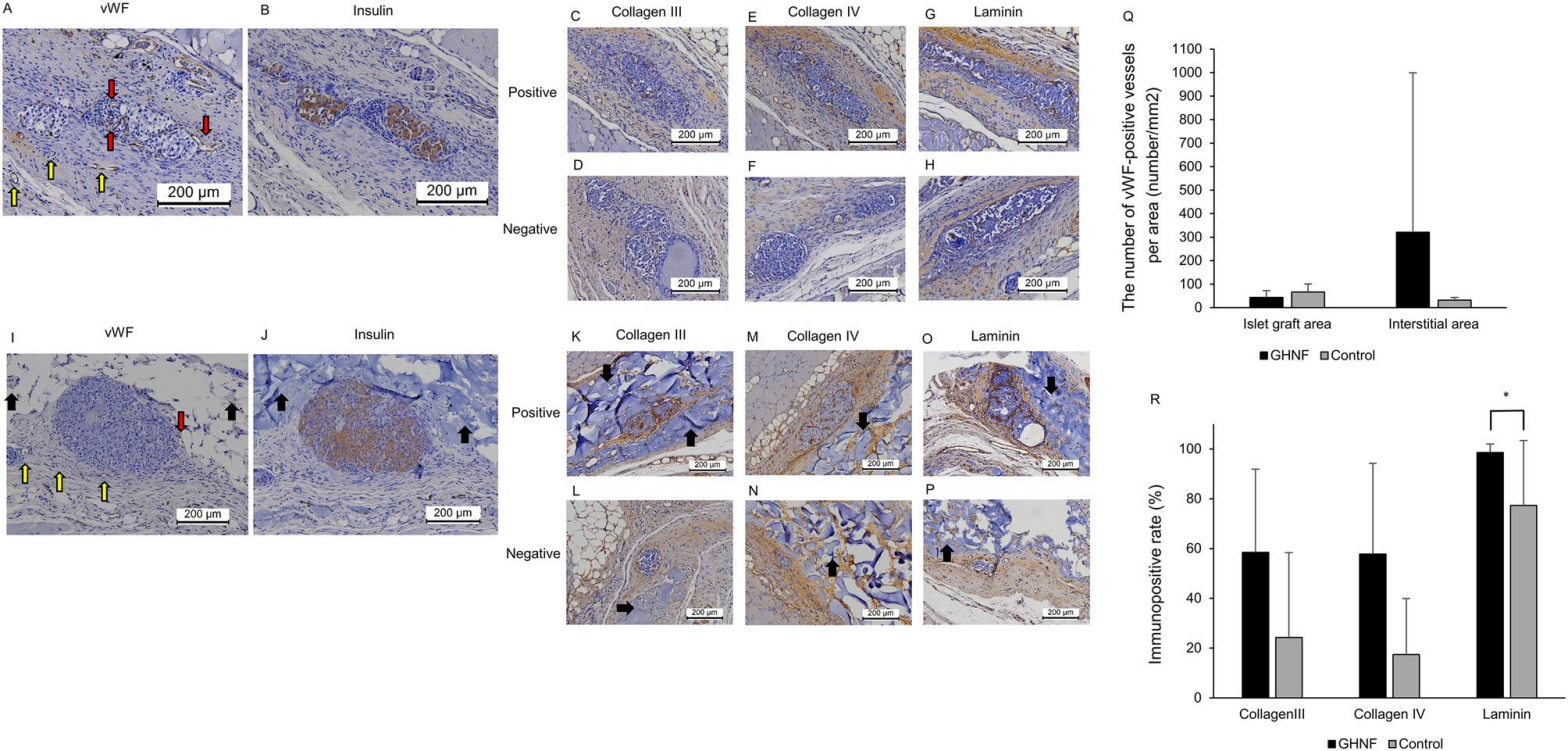
Immunohistochemical analyses. (**A**–**H**: Control group, **I**–**P**: GHNF group) Representative photomicrographs of von Willebrand factor (vWF), insulin, collagen III, collagen IV, and laminin staining. The vWF-positive vessels were detectable in the fibrous capsule around the islets (red arrows). New vessels in the interstitial area are also represented by yellow arrows. GHNF was represented by black arrows. “Positive” for collagen III, collagen IV, and laminin indicates that distinct immunopositivity was detectable in the fibrous capsule around the islets. “Negative” indicates that immunopositivity was undetectable. Magnification: × 200. Calibration bars: 200 µm. (Q) The mean number of new vessels per islet graft area and per interstitial area. No significant difference was observed between the GHNF and control groups in the islet graft area. In the interstitial area, the number of new vessels in the GHNF group tended to be higher in comparison to that in the control group (*p* = 0.06). (R) The rate of immunopositive sections in terms of collagen III, collagen IV, and laminin in the fibrous tissue around the islet grafts. The rate of laminin positivity in the GHNF group was significantly higher than that of the control group (*p < 0.05). The rate of collagen III and collagen IV positivity in the GHNF group also tended to be higher in comparison to the control group (*p* = 0.07, 0.06, respectively).

The number of anti-von Willebrand factor (vWF) positive vessels surrounding islets was counted to examine the state of neovascularization of the islet grafts (Fig. 4A,I). No significant difference was observed between the GHNF and control groups (Fig. 4Q). On the other hand, in the interstitial area, the number of new vessels in the GHNF group tended to be higher in comparison to the control group, but it did not reach statistical significance (*p* = 0.064) (Fig. 4Q). The insulin staining was also performed in both groups (Fig. 4B,J). The insulin-positive area appeared to be larger in the GHNF group, although no quantitative evaluation was performed in the present study. To examine the effect of ECM on subcutaneous islet engraftment, the rate of immunopositive sections, in terms of collagen III, collagen IV and laminin at the fibrous capsule around the islet grafts was evaluated (Fig. 4C–H,K–P). The rate of laminin positivity in the GHNF group was significantly higher than that in the control group (*p* = 0.030) (Fig. 4R). The rates of collagen III and collagen IV positivity in the GHNF group also tended to be higher in comparison to the control group; however, the difference did not reach statistical significance (*p* = 0.068, *p* = 0.059, respectively) (Fig. 4R).

### Quantification of the vascular volume using CT angiography

**Figure 5:**
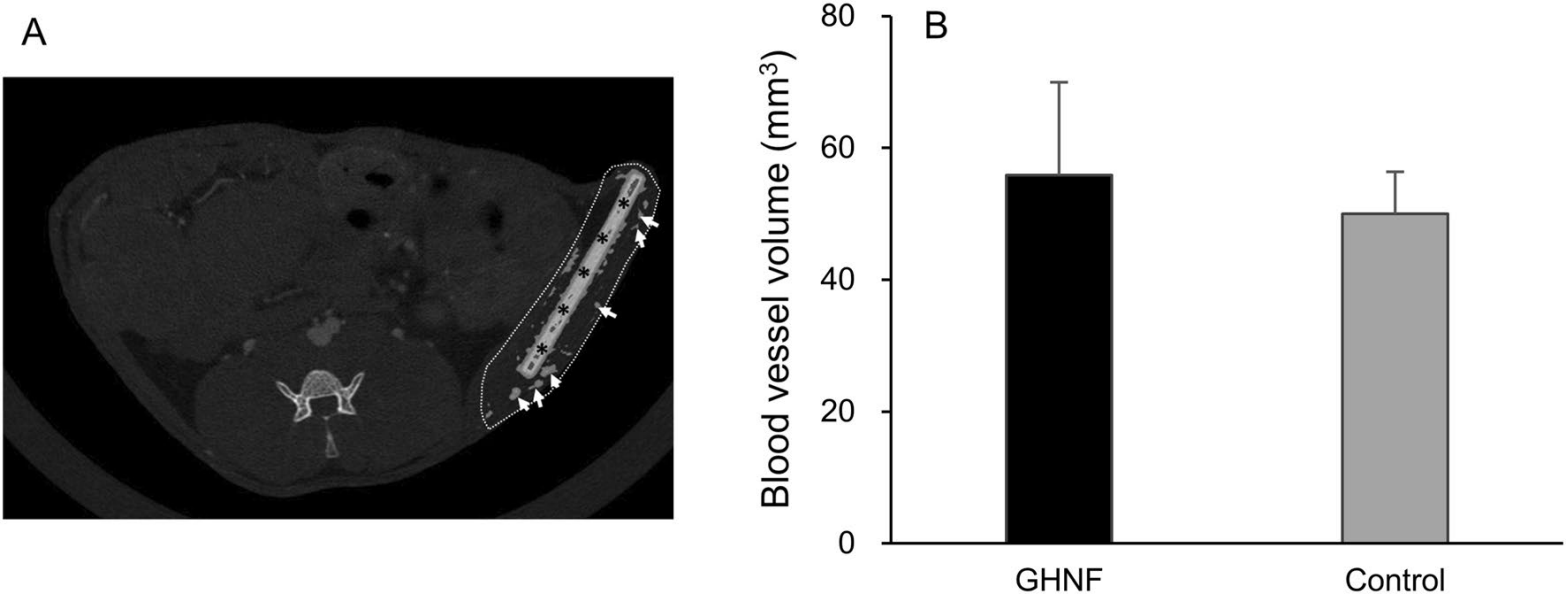
Quantification of the vascular volume determined through CT analyses in subcutaneous tissues at 6 weeks after pretreatment. (**A**) A representative CT angiography image. Special care was taken in defining the region of interest (inside dot line) to avoid inclusion of intraperitoneal vascular area. The extracted area around silicon spacer (asterisk) was defined as a vascular area (white arrow). (**B**) The vascular volume in the GHNF and control groups. The results represent the volume (mm^3^) and are expressed as the mean ± standard deviation (n = 6).

CT angiography revealed that the blood vessel volume surrounding the silicon spacer was comparable between the GHNF and control groups (54.5 ± 11.9 mm^3^ vs. 51.8 ± 8.6 mm^3^, *p* = 0.687) (Fig. 5A,B).

### The relative gene expression

**Figure 6:**
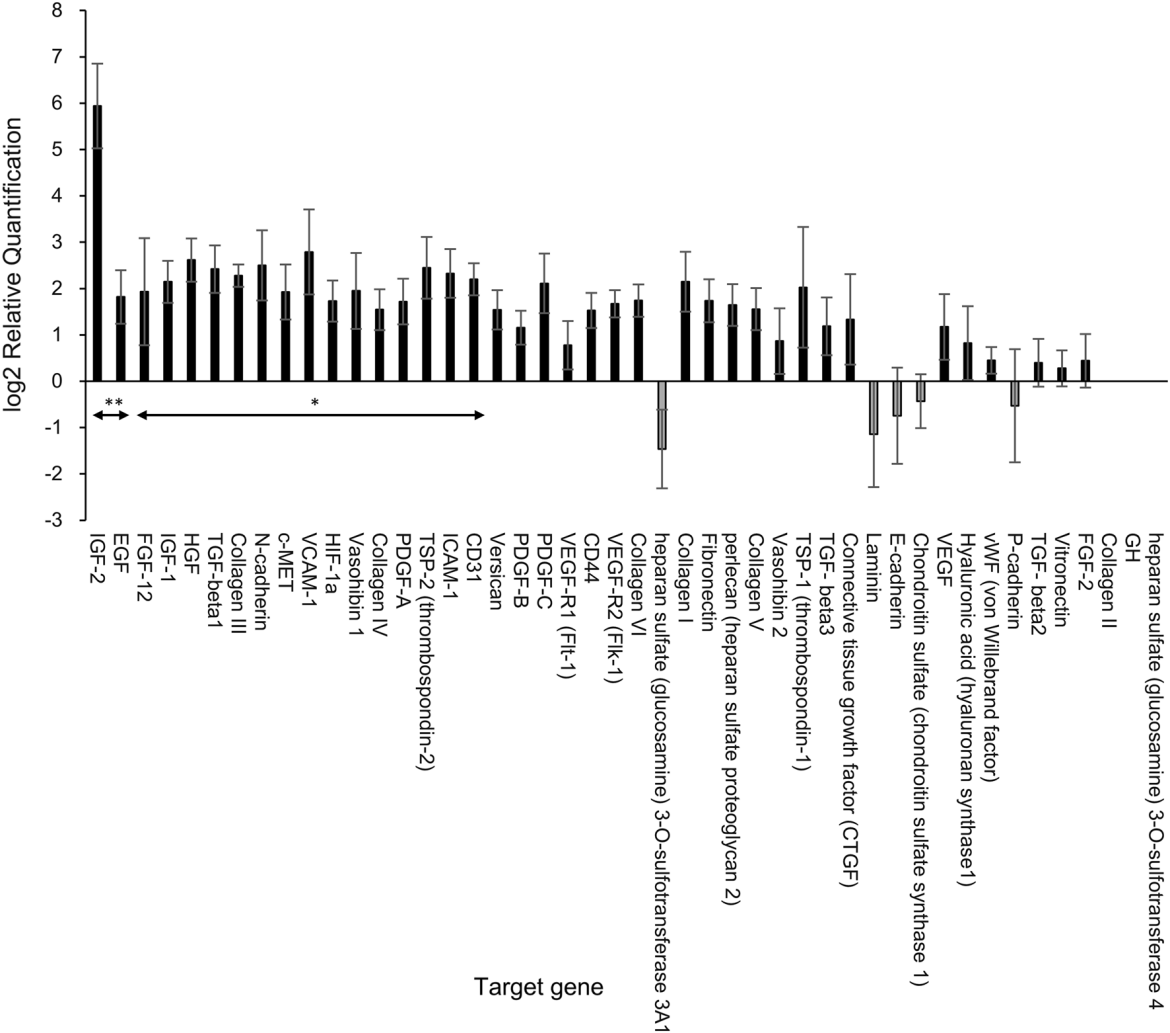
Upregulated and downregulated genes in the GHNF group (n = 8) in comparison to the control group (n = 8). RNA was extracted from the recipient subcutaneous capsules surrounding the silicon spacer at 42 days after pretreatment. Values represent the mean relative quantification (RQ). Error bars represent the standard error on a log2 RQ-based scale. The + 1 and − 1 values represent a two-fold increase or decrease threshold in the gene expression. The gene expression analyses showed that 19 target genes in the GHNF group were significantly upregulated in comparison to the control group (*p < 0.05, **p < 0.01).

In order to identify genes that were upregulated in the subcutaneous capsules in the GHNF group, 46 target genes were analyzed using TaqMan Arrays (Table 1). The relative gene expression in the GHNF group is shown in Fig. 6. The gene expression analyses revealed that 19 target genes (insulin-like growth factor 2 (IGF-2), epidermal growth factor (EGF), fibroblast growth factor 12, insulin-like growth factor 1 (IGF-1), hepatocyte growth factor (HGF), transforming growth factor, beta 1 (TGF-β1), collagen III, N-cadherin, mesenchymal-epithelial transition factor (c-MET), vascular cell adhesion molecule-1 (VCAM-1), hypoxia inducible factor 1 alpha subunit (HIF-1α), Vasohibin 1, collagen IV, platelet derived growth factor, alpha (PDGF-A), thrombospondin 2 (TSP-2), intercellular adhesion molecule 1 (ICAM-1), CD31, Versican and platelet derived growth factor, B polypeptide (PDGF-B)) were significantly upregulated in comparison to the control group (*p* < 0.05). Of particular interest, IGF-2 was remarkably upregulated (The relative quantification (RQ); 61.74). In this assay, the islet-derived factors were not included since the tissues were analyzed prior to islet transplantation.

**Table 1:** The list of analyzed target genes.

Assay ID	Gene symbol	Gene name
Hs99999901_s1	18s rRNA	18S ribosomal RNA
Mm99999915_g1	Gapdh	Glyceraldehyde-3-phosohate dehydrogenase
Mm00550376_m1	Vwf	Von Willebrand factor homolog
Mm01242576_m1	Pecam1	Platelet/endothelial cell adhesion molecule 1
Mm01226102_m1	Lama1	Laminin, alpha 1
Mm01256744_m1	Fn1	Fibronectin 1
Mm00495976_m1	Vtn	Vitronectin
Mm03649146_m1	Hs3st4	Heparan sulfate (glucosamine) 3-O-sulfotransferase 4
Mm01181173_g1	Hspg2	Perlecan (heparan sulfate proteoglycan 2)
Mm00780907_s1	Hs3st3a1	Heparan sulfate (glucosamine) 3-O-sulfotransferase 3A1
Mm00801666_g1	Col1a1	Collagen, type I, alpha 1
Mm01309565_m1	Col2a1	Collagen, type II, alpha 1
Mm00802300_m1	Col3a1	Collagen, type III, alpha 1
Mm01210125_m1	Col4a1	Collagen, type IV, alpha 1
Mm00489299_m1	Col5a1	Collagen, type V, alpha 1
Mm00487160_m1	Col6a1	Collagen, type VI, alpha 1
Mm01247357_m1	Cdh1	Cadherin 1
Mm01162497_m1	Cdh2	Cadherin 2
Mm01249209_m1	Cdh3	Cadherin 3
Mm00516023_m1	Icam1	Intercellular adhesion molecule 1
Mm01320970_m1	Vcam1	Vascular cell adhesion molecule 1
Mm01135184_m1	Hgf	Hepatocyte growth factor
Mm00437306_m1	Vegfa	Vascular endothelial growth factor A
Mm00438980_m1	Flt1	FMS-like tyrosine kinase 1
Mm01222421_m1	Kdr	Kinase insert domain protein receptor
Mm00439560_m1	Igf1	Insulin-like growth factor 1
Mm00439564_m1	Igf2	Insulin-like growth factor 2
Mm00438696_m1	Egf	Epidermal growth factor
Mm00433590_g1	Gh	Growth hormone
Mm01205760_m1	pdgfa	Platelet derived growth factor, alpha
Mm00440677_m1	pdgfb	Platelet derived growth factor, B polypeptide
Mm00480205_m1	pdgfc	Platelet derived growth factor, C polypeptide
Mm01285715_m1	Fgf2	Fibroblast growth factor 2
Mm00679872_m1	Fgf12	Fibroblast growth factor 12
Mm01156972_m1	Met	Met proto-oncogene
Mm00468869_m1	Hif1a	Hypoxia inducible factor 1, alpha subunit
Mm00449032_g1	Thbs1	Thrombospondin 1
Mm01279240_m1	Thbs2	Thrombospondin 2
Mm01178820_m1	Tgfb1	Transforming growth factor, beta 1
Mm00436955_m1	Tgfb2	Transforming growth factor, beta 2
Mm00436960_m1	Tgfb3	Transforming growth factor, beta 3
Mm00616592_m1	Vash1	Vasohibin 1
Mm00523123_m1	Vash2	Vasohibin 2
Mm03048195_m1	Has1	Hyaluronan synthase 1
Mm05751874_s1	Chsy1	Chondroitin sulfate synthase 1
Mm01283063_m1	Vcan	Versican
Mm01277161_m1	Cd44	CD44 antigen
Mm01192933_g1	Ctgf	Connective tissue growth factor

### The IGF-2 immunoassay

**Figure 7:**
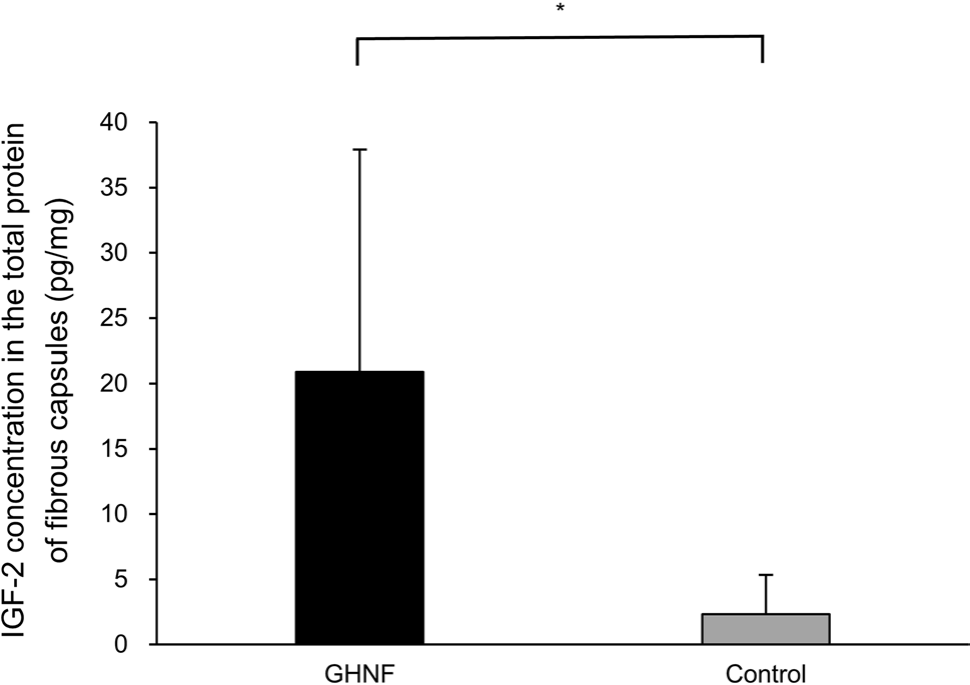
The concentration of IGF-2 in the total protein of the homogenized subcutaneous fibrous capsules surrounding the silicon spacer at 6 weeks after pretreatment (GHNF; n = 5, control; n = 6). The concentration of IGF-2 in the GHNF group was significantly higher than that in the control group (*p < 0.05).

The concentration of IGF-2 in the total protein of the homogenized subcutaneous fibrous capsules in the GHNF group was significantly higher than that in the control group (20.91 ± 17.02 pg/mg vs. 2.33 ± 3.00 pg/mg, *p* = 0.02) (Fig. 7).

## Discussion

The present study showed that pretreatment of a transplant site using a gelatin hydrogel nonwoven fabric (GHNF) markedly improved the outcomes of subcutaneous islet transplantation. To our knowledge, this is the first report to demonstrate that the outcomes of subcutaneous islet transplantation are apparently superior to those of a current gold standard, intraportal transplantation, when the same amounts of islets are implanted. In fact, the cure rate of diabetic mice in the Ipo group (35.7%) was better than that of the unmodified subcutaneous transplant group (without GHNF) (22.7%). In sharp contrast, all diabetic mice were cured in the GHNF-treated subcutaneous transplant group. Likewise, exactly same tendency was observed in the glucose tolerance of the three groups. Of particular note, the GHNF group demonstrated not only a higher cure rate of diabetic animals, but also a substantial short period to the achievement of euglycemia in comparison to the Ipo group. Considering that it usually takes long time for transplanted islet grafts to function in the subcutaneous space, the present novel procedure can make up for this drawback, and may therefore be regarded as more practical for clinical use.

In addition, unlike basic fibroblast growth factor^17,21^, no bleeding or inflammation were detected at the transplant site in the GHNF group. This feature of GHNF may also be desirable for clinical application.

In the present study, the immunohistochemical analyses showed that the number of vWF-positive vessels surrounding islets were comparable between the GHNF and control groups. Corroborating this finding, CT angiography also revealed that the blood vessel volume surrounding the silicon spacer was equivalent between both groups. In light of several previous reports showing that a main drawback of subcutaneous islet transplantation is the poor neovascularization of islet grafts^12,13^, the abovementioned findings of the present study were unexpected. Given that islet neovascularization was reported to be extremely low and insufficient, even in intraportal transplantation^22,23^, these unexpected findings suggest that crucial factors, other than neovascularization, may contribute to the beneficial effects of GHNF. Moreover, these findings also suggest that a combination strategy that involves the use of GHNF and adipose tissue-derived stem cells, which is well known to facilitate islet revascularization^24^, may be useful for further prolonging graft survival in the subcutaneous space.

One possible candidate factor for the beneficial effects of GHNF is the compensation of ECM, which was reported to be lost from the capsule surrounding the islets during the islet isolation procedure^25^. Indeed, the immunohistochemical analyses in this study demonstrated that the expression levels of laminin, collagen III and collagen IV in the fibrous capsule surrounding the islet grafts were enhanced in the GHNF group in comparison to the control group. These findings are consistent with several previous studies that reported that the compensation of ECM surrounding the islet grafts is closely related to the islet function^26–31^. Corroborating the above-noted findings, the gene expression levels of collagen III and collagen IV were also significantly upregulated in the GHNF group. In addition, the gene expression of TGF-β1, which is known to closely related to the production of several types of ECM, was also upregulated in the GHNF group. Interestingly, Lee et al.^32^ previously reported that TGF-β1 in combination with IGF-1, HGF and transforming growth factor alpha (TGF-α), could improve the islet function. In support of this report, in addition to TGF-β1, IGF-1 and HGF were upregulated in the GHNF group in the present study, suggesting that these factors may synergistically act on improving the islet function.

Another possible candidate for crucial factors of GHNF is the protective effect of growth factors. In the present study, the gene expression analyses revealed that various growth factors were in fact significantly upregulated in the GHNF group in comparison to the control group. In particular, IGF-2 was markedly upregulated. Furthermore, not only the gene expression but also the protein levels of IGF-2 were significantly upregulated in the GHNF group. In accordance with our findings, IGF-2 has been reported to maintain islet viability during the culture period, and to improve islet engraftment due to its potent anti-apoptotic effect^33–38^. Furthermore, it was reported that in addition to the anti-apoptotic effects of IGF-2 on islets, its actions can be observed in various other cell and tissue types, including neurons and ovarian pre-ovulatory cells^39^. Of particular interest, Hughes et al. previously reported that the development of novel biomaterials coated with IGF-2 is a promising strategy to improve islet engraftment^40^. We believe that GHNF corresponds exactly to this novel biomaterial. Likewise, EGF, IGF-1, HGF and N-cadherin were also reported to improve islet engraftment with effects that are similar to those of IGF-2^41–46^. In light of the outcome of the gene expression analyses in the present study, these factors may have also contributed to the beneficial effects of GHNF.

The TaqMan Array analyses in this study also revealed the upregulation of HIF-1α in the GHNF group. Considering the severe hypoxic milieu in the subcutaneous tissues, the upregulation of HIF-1α, irrespective of GHNF pretreatment, was expected and logical. However, this cannot explain why HIF-1α was only significantly upregulated in the GHNF group. Notably, Feldser et al.^47^ previously reported that IGF-2 itself can efficiently induce HIF-1α, and vice versa. In support of this finding, Pringle et al.^48^ reported that complex cross-talk between IGF-2 and HIF-1α is of importance in the regulation of trophoblast behavior. Based on these reports, it seems more likely that HIF-1α in the GHNF group was upregulated by not only hypoxic conditions but also by complex interactions with abundant IGF-2.

In the present study, we transplanted 400 islet equivalents (IEQs) of islets to the recipients as the marginal mass according to our previous report^17^. The cure rate of the Ipo group in the present study (35.7%) was consistent with previous studies^49,50^ (44.4% and 47.7%, respectively). However, Cui et al.^51^ reported that 250 islets (not IEQs) were enough to cure 46.7% of diabetic mice, while Alwahsh et al.^52^ reported that no diabetic mice were cured by 400 islets (not IEQs). One possible explanation for this discrepancy may be based on ambiguity of the islet selection procedure. Considering that an islet with a 150-µm diameter corresponds to 1 IEQ, if researchers selected islets with an average diameter of 170 µm in their study, 250 islets would be equivalent to 364 IEQs. Since the islet number method is imprecise, we introduced the islet IEQ method in order to determine the marginal mass in the present study. However, regardless of the islet counting method, this study clearly revealed that subcutaneous transplantation with GHNF pretreatment was more efficient than intraportal transplantation by transplanting exactly the same amount of islets to both sites.

Although the GHNF does not have lot-to-lot variation problems, the indwelling period of this type of material may vary depending on the animal species. Thus, further studies using large animal models are warranted as steps toward practical use.

In conclusion, the present study showed that pre-implantation of a novel gelatin hydrogel nonwoven fabric effectively improved subcutaneous islet engraftment, resulting in much better outcomes than intraportal islet transplantation. This beneficial effect may be mainly due to the compensation of ECM for the islet capsule and protection of islet viability by various growth factors, rather than the enhancement of neovascularization.

## Materials and methods

### Animals

All animals in the present study were handled in accordance with the Animal Research: Reporting of In Vivo Experiments (ARRIVE) guidelines, the Guide for the Care and Use of Laboratory Animals published by the National Institutes of Health^53^. All experimental protocols of the present study (protocol ID: 2018 MdA-175) were approved by the animal experimental committee in the Tohoku University. C57BL/6 mice (male; age, 8–14 weeks) (Japan SLC, Inc., Shizuoka, Japan) were used as both donors and recipients. All mice were housed under specific pathogen-free conditions and had free access to food and water. All surgeries were performed under general anesthesia using isoflurane, and all efforts were made to minimize suffering.

### The induction and diagnosis of diabetes in the recipients

Diabetes was induced by the intravenous injection of 170 mg/kg streptozotocin (STZ) (SIGMA-ALDRICH, Inc., MO, USA). Mice whose non-fasting blood glucose levels were ≥ 400 mg/dL on two consecutive measurements were considered diabetic. Serial blood glucose levels were determined, and the recipients whose non-fasting blood glucose levels were < 200 mg/dL on two consecutive measurements were considered to be cured.

### Islet isolation

Islet isolation and culturing were performed as previously described^14,54^. In brief, the bile duct was identified and clamped at the papilla of Vater. Two milliliters of cold Hank’s^12^ balanced salt solution (HBSS) containing 1 mg/mL collagenase (Sigma type V; Sigma Chemicals, St. Louis, MO, USA) was injected into the common bile duct leading to the pancreas under a stereomicroscope. The pancreas was removed and incubated in a water bath at 37 °C for 12 min before being digested, and the cell suspension was washed 3 times in cold HBSS and centrifuged for 1 min. Density-gradient centrifugation was performed for 10 min using a Histopaque-1119 (Sigma Diagnostics, St. Louis, MO, USA) and Lymphoprep™ (Axis-Shiled, Oslo, Norway) to isolate pancreatic islets. The islets were cultured in Roswell Park Memorial Institute-1640 medium containing 5.5 mmol/L glucose and 10% fetal bovine serum at 37 °C in 5% CO_2_ and humidified air overnight before transplantation.

### Implantation of a gelatin hydrogel nonwoven fabric and islet transplantation

A gelatin hydrogel nonwoven fabric (Genocel; NIKKE MEDICAL Co., Ltd., Osaka, Japan) was prepared using a previously reported method^18^, and processed into a circular sheet type (11 mm diameter, 0.5 mm thickness). After swelling with physiological saline, two GHNF sheets sandwiching a silicon spacer (11 mm diameter, 0.5 mm thickness) were placed into the left dorsal subcutaneous space at 6 weeks before islet transplantation (GHNF group). In the control group, a silicon spacer without GHNF was placed into the left dorsal subcutaneous space at 6 weeks before islet transplantation. GHNF was implanted into healthy mice, then the mice were injected with STZ, 7 days before islet transplantation.

After the removal of the silicon spacer (Fig. 1A,B), 400 IEQs (approximately 20,000 IEQs/kg) of syngeneic mouse islets were transplanted into the pretreated space using a gastight syringe (Hamilton Co., Reno, NV, USA)^17^ in the GHNF and control groups. On the other hand, in the intraportal transplantation (Ipo) group, 400 IEQs of syngeneic mouse islets in a total volume of 300 µL were infused into the recipient liver through the portal vein using a 27-gauge Surshield (TERUMO, Inc., Tokyo, Japan)^55^. The recipients were followed by measuring non-fasting blood glucose levels every 3–4 days throughout the study period (42 days after islet transplantation) (Fig. 1C).

### Intraperitoneal glucose tolerance test

An IPGTT was performed 43–47 days after islet transplantation, as described previously^56^. In brief, recipients that had been fasted for 14 h were intraperitoneally infused with D-glucose (1.0 g/kg), and the blood glucose concentrations were determined before and at 5, 10, 15, 20, 25, 30, 45, 60, 90, and 120 min after the injection of glucose. Then, the blood glucose curve was generated, and the AUC was used for comparison.

### Immunohistochemical analyses

At 7 days after transplantation, the recipient tissues at the site of subcutaneous islet transplantation were procured, fixed with 4% paraformaldehyde, and embedded in paraffin for immunohistochemical staining. Immunohistochemical staining was performed using an vWF (ab7356; MerckMillipore, Darmstadt, Germany), anti-insulin (Dako, Glostrup, Denmark), anti-collagen III (ab7778; Abcam, Cambridge, UK), anti-collagen IV (ab6586; Abcam) and anti-laminin (ab11575; Abcam) antibodies. EnVision+ System- HRP labelled polymer anti-rabbit (4003; DAKO) was used as a secondary antibody. For the evaluation of neovascularization, the vWF-positive cells in the islet and interstitial areas were counted^57^. In collagen III^58^, collagen IV and laminin staining, “positive” was defined as marked immunopositivity that was detectable in the fibrous capsule around the islets. More than 4 sections with almost the same intervals from each experimental group (GHNF; n = 7, control; n = 8) were evaluated by a pathologist using a blind method.

### Computed tomography angiography

CT angiography was performed in the GHNF (n = 6) and control (n = 6) groups at 6 weeks after pretreatment (Fig. 5A). Before CT imaging, 8 µl/g body weight of ExiTron nano 12,000 (Miltenyi Biotec, Bergisch Gladbach, Germany) was injected intravenously via the tail vein. ExiTron nano 12,000 is a high-density, alkaline earth metal-based nanoparticulate contrast agent that was specifically formulated for animal CT. These nanoparticles have a mean diameter of 110 nm, suggesting that 110 nm (in diameter) is a threshold of detectability for the size of new vessels. CT angiography was performed using an X-ray CT scanner for experimental animals (Latheta LCT-200; FUJIFILM Healthcare Manufacturing Corporation, Chiba, Japan), and blood vessel volume was calculated using attached software in the Latheta LCT-200. The vascular volume in subcutaneous tissues surrounding the silicon spacer was calculated. The CT value of the vascular area was defined as > 100, in order to extract small blood vessels.

### Real-time PCR using TaqMan arrays

RNA was extracted from the recipient subcutaneous fibrous capsules surrounding the silicon spacer at 6 weeks after pretreatment (GHNF; n = 8, control; n = 8)^59^. The relative gene expression was determined using a TaqMan array 96-well FAST plate (4,413,257, Applied Biosystems; Bedford, Massachusetts, US). A TaqMan array plate contains 46 target genes and two assays for candidate endogenous control genes (Table 1). The samples were analyzed using a StepOnePlus Real-Time polymerase chain reaction (PCR) System (Applied Biosystems) under the following amplification conditions: 50 °C for 2 min and 95 °C for 20 s, followed by 40 cycles of 95 °C for 1 s and 60 °C for 20 s. The results were analyzed using ExpressionSuite Software ver. 1.3 (Applied Biosystems). RQ was calculated using the comparative CT method. In order to determine the relative gene expression in the GHNF group, the samples in the control group were designated as a calibrator. 18S was used as housekeeping gene.

### An IGF-2 immunoassay using an enzyme-linked immunosorbent assay (ELISA)

The recipient subcutaneous fibrous capsule surrounding the silicon spacer at 6 weeks after pretreatment was procured (GHNF; n = 5, control; n = 6). The recipient subcutaneous fibrous capsules were homogenized using SONICS Vibra-cell (SONICS & MATERIALS, Inc., Newtown, CT, USA) in 5 volumes of RIPA buffer (50 mM Tris–HCl, 150 mM NaCl, 1 mM ethylenediaminetetraacetic acid, 1.0% NP-40, 0.5% sodium deoxycholate, 0.1% sodium dodecyl sulfate) (FUJIFILM Wako Pure Chemical Corporation, Osaka, Japan) supplemented with 1 mM phenylmethylsulfonyl fluoride (PMSF) (SIGMA-ALDRICH, Inc.) and 1.0% Protease Inhibitor Cocktail Set V (FUJIFILM Wako Pure Chemical Corporation). The extract was centrifuged at 10,000 g for 20 min, and then the supernatant was recovered, diluted, aliquoted and frozen at − 20 to − 80 °C. The concentrations of total protein and IGF-2 in the supernatant were measured using a Pierce BCA Protein Assay kit (Thermo Fisher Scientific Inc., Waltham, MA, USA) and a Quantikine ELISA Mouse/Rat/Porcine/Canine IGF-2 Immunoassay Kit (R&D Systems, Minneapolis, MN, USA) according to the manufacturer’s instructions.

### Statistical analyses

All data are expressed as the mean ± standard deviation. All statistical analyses were performed using the JMP pro 15 software program (SAS Institute Inc., Cary, NC, USA). The changes in the blood glucose levels and IPGTT were analyzed by a two-way analysis of variance (ANOVA) and a Tukey–Kramer test was used for post-hoc comparisons between the groups. The AUC of the IPGTT was analyzed by a Kruskal–Wallis test, followed by Dunn’s post-hoc test. The number of vWF-positive vessels, the immunopositive rate in ECM staining, and IGF-2 Immunoassay were analyzed by a Mann Whitney U test. Kaplan–Meier curves were compared using a log-rank test. P values of < 0.05 were considered to indicate statistical significance.

## Data Availability

All data generated or analyzed in the present study were included in this published manuscript.

## Abbreviations

ANOVA: Analysis of variance
AUC: Area under the curve
CT: Computed tomography
ECM: Extracellular matrices
EGF: Epidermal growth factor
HBSS: Hank’s balanced salt solution
HGF: Hepatocyte growth factor
HIF-1α: Hypoxia inducible factor 1 alpha subunit
IEQs: Islet equivalents
IGF-1: Insulin-like growth factor 1
IGF-2: Insulin-like growth factor 2
IPGTT: Intraperitoneal glucose tolerance test
Ipo: Intraportal
PCR: Polymerase chain reaction
RCP: Recombinant peptide
RQ: Relative quantification
STZ: Streptozotocin
TGF-α: Transforming growth factor, alpha
TGF-β1: Transforming growth factor, beta 1
vWF: Von Willebrand factor
